# Physiotherapy Intervention in a Patient With a Glioma of the Cingulate Gyrus: A Report of a Rare Case

**DOI:** 10.7759/cureus.54385

**Published:** 2024-02-18

**Authors:** Vishal U Telrandhe, H V Sharath, Ruchika J Zade

**Affiliations:** 1 Department of Pediatric Physiotherapy, Ravi Nair Physiotherapy College, Datta Meghe Institute of Higher Education & Research, Wardha, IND; 2 Department of Neurophysiotherapy, Ravi Nair Physiotherapy College, Datta Meghe Institute of Higher Education & Research, Wardha, IND

**Keywords:** diminished vision, multimodal stimulation, postoperative physiotherapy, cingulate gyrus, glioma

## Abstract

The cortical part of the limbic system, the cingulate gyrus (CG), is a conspicuous structure present in the central aspect of the cerebral cortex. It is associated with various cognitive, emotional, and behavioral purposes and has a significant impact on the limbic system, which is responsible for emotions and memory processes. The aim of the study is to comprehensively document and evaluate the effectiveness of physiotherapy intervention in managing a rare case of glioma specifically located in the CG through a concise and impactful online presentation. A CG lesion refers to damage or injury to the CG, a part of the brain located in the cerebral cortex’s medial (inner) aspect. A 45-year-old female was admitted to the neurosurgery ICU with a complaint of diminished vision and headaches for the past 1.5 months. She had a history of fever and vomiting. She was diagnosed with a CG lesion on the contrast-enhanced MRI of the brain. This report of a rare case highlights the potential benefits of physiotherapy intervention in a patient with a glioma of the CG. The unique challenges posed by this specific brain tumor location necessitate a tailored and multidisciplinary approach to patient care.

## Introduction

Cingulate gyrus (CG) epilepsies were defined as complex partial seizures with complicated motor gestural automatisms at onset, autonomic symptoms, and alterations in mood and affect. The CG, positioned medially in each hemisphere, is the biggest and most recently formed limbic structure. The cingulate is involved in a variety of activities, including cognition and emotion integration, regulation of emotion-related autonomic activity, and emotional reactions to pain. In addition, it has been demonstrated that the cingulate is involved in attention, executive functions, word production, and memory. The cortical part of the limbic system consists of the CG and the substantia grisea, which line the superior and inferior margins of the pars marginalis [1].

It originates under the corpus callosum’s (CC) rostrum, wraps around the genu, and projects over the superior surface of the CC’s body, attaining its posterior terminus at the isthmus of the CG. In the temporal lobe, the isthmus connects to the parahippocampal gyrus. The callosal sulcus segregates the CG from the CC inferiorly, while it separates it from the superior frontal gyrus superiorly [2-3]. The CG is part of the mesocortex, commonly referred to as the juxtallocortex. It develops along the margins of the cerebral cortex’s isocortex and allocortex-like transitional zones [4,5]. Previous gross anatomical and functional imaging investigations indicated the existence of underlying subcortical fibers that link the anterior CG to the frontal cortex or the posterior CG to the parietal cortex [6,7]. This case report aims to shed light on the nuanced physiotherapy interventions employed in a patient diagnosed with glioma of the CG. The rarity of such cases underscores the need for a comprehensive understanding of the impact of gliomas on motor and functional abilities, as well as the potential role of physiotherapy in optimizing patient outcomes. By examining the challenges faced and strategies implemented in this unique case, we hope to contribute valuable insights to the evolving landscape of neurorehabilitative care for individuals confronting gliomas in this anatomically critical region [8,9].

The anterior cingulate cortex, midcingulate cortex, posterior cingulate cortex, and retrosplenial cortex are the four functionally different sections of the cingulate cortex [10]. CG epilepsy is debatable since it might have separate clinical characteristics or overlap with other frontal lobe epilepsy types. Andy and Chinn used the term anterior cingulate epilepsy to illustrate an experimental epilepsy model in cats in 1957 [11]. Mazars published “Criteria for identifying cingulate epilepsies” in 1970, including 36 instances [12]. This work preceded MRI and CT, and intracerebral depth electrode mapping was difficult by today’s standards [13]. Cingulate epilepsy was recognized as a kind of frontal lobe epilepsy in the suggested taxonomy of epilepsy and epileptic syndromes by the International League Against Epilepsy in 1989 [14]. The commission initially outlined overarching characteristics that strongly indicate a diagnosis of frontal epilepsy: short seizures, minimal or no postictal confusion, rapid progression to secondary generalized seizures, prominent motor manifestations, complex gestural automatisms, and frequent falling when the discharge is bilateral. Subsequent to that, it gave a concise description of seizure subtypes.

CG gliomas, tumors located within the CG of the brain, often pose challenges to individuals due to their impact on motor function, coordination, and overall physical well-being. Physiotherapy emerges as a valuable adjunctive treatment approach for managing these challenges [15-17]. Physiotherapists design tailored rehabilitation programs to address specific motor deficits associated with CG gliomas, incorporating exercises that enhance muscle strength, coordination, and balance. These interventions aim to improve the individual’s overall mobility and reduce functional limitations resulting from the tumor. Additionally, physiotherapy plays a role in pain management, employing strategies such as targeted exercises, manual therapy, and modalities to alleviate discomfort associated with the tumor or its treatment. By focusing on individualized treatment plans and collaborating with multidisciplinary teams, physiotherapists contribute significantly to enhancing the quality of life and functional independence of individuals facing CG gliomas [18-20].

## Case presentation

Medical history 

The patient was a 45-year-old female who arrived at the casualty department via ambulance with symptoms of diminished vision in the bilateral eye, headache, seizures (bilateral asymmetrical tonic seizure), vomiting, and no known underlying conditions. She had been experiencing headaches and seizures for two months. Additionally, she had reported a history of fever and reduced appetite for three days. Furthermore, she was suffering from vomiting episodes occurring shortly after food intake, a concern that has been present for the past day, for which she approached our tertiary care for additional treatment. Crucially, the patient has no history of hypertension, diabetes, or any other comorbidities, including head injury and loss of consciousness. After consultation, she was advised to undergo a CT/MRI of the brain and a two-dimensional echocardiography. She was diagnosed with a CG space-occupying lesion. Under general anesthesia, the head was in a neutral position. The incision was marked under navigation guidance. Parts were painted and draped in a linear incision. A 3 × 3 cm parasagittal craniotomy was performed. Dura reflected medially. An interhemispheric approach was taken to the tumor. It was grayish soft, mildly vascular, with no clear plane of cleavage from the brain parenchyma. Decompression was done, and hemostasis was achieved. Dura closed the water tightly, the bone flap was replaced, and it was secured with two plates and four screws. The wound was closed in layers, and the sterile dressing was done on August 29, 2023. On postoperative Day 1, she was transferred to the neuro-ICU and assigned to neurophysiotherapy.

Clinical findings

On Observation

The patient was seen in a supine lying position with the head end elevated at a 35-degree angle. Her physique exhibited a mesomorphic body type, maintaining a BMI of 22 kg/m2 within the normal range. On neurological examination, the Glasgow Coma Scale (GCS) score indicated moderate impairment (E3V1M5). Initially, the sensations were not assessable. Superficial sensations were intact, deep and cortical sensations were not assessable, and all the cranial nerves were intact except the second (optic nerve). On motor examination, tone was evaluated on the Tone Grading Scale; initially, it was hypotonia and flaccid on the affected side (Table 1). Muscle strength was evaluated on a Voluntary Control Grading Scale (Table 2). Superficial and deep reflexes were assessed (Table 3). The coordination test and gait were not assessable.

**Table 1:** Muscle tone on TGS TGS, Tone Grading Scale

	Upper limb	Lower limb
Right	1+ (hypotonia)	0 (flaccid)
Left	2+	2+

**Table 2:** Voluntary Control Grading Scale

	Upper limb	Lower limb
Right	1	0
Left	6	6

**Table 3:** Reflexes

	Biceps jerk	Triceps jerk	Supinator jerk	Knee jerk	Ankle jerk	Plantar response
Right	1+	1+	1+	1+	Absent	Absent
Left	2+	2+	2+	2+	2+	Flexor

Diagnostic assessment

**Figure 1:**
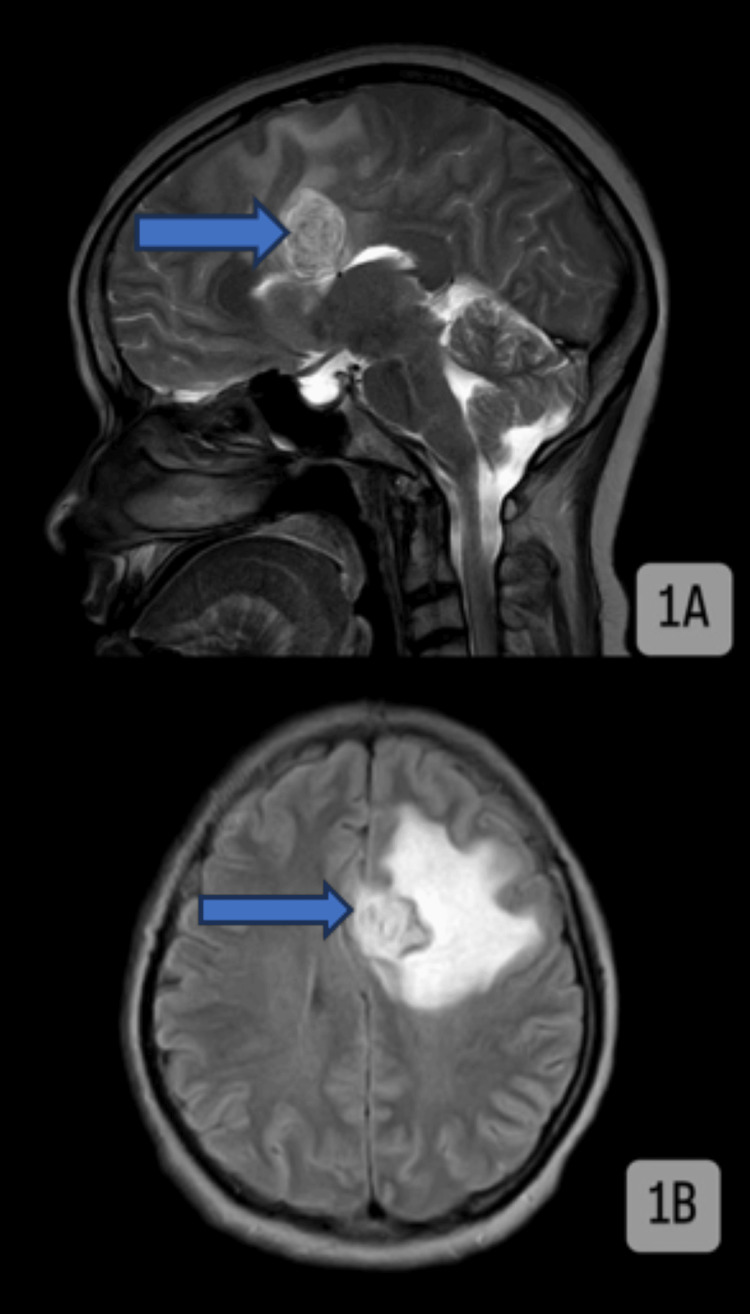
MRI of the brain (1A) Heterogeneously intensely enhancing altered signal intensity of CG appearing heterogeneously hyperintensity on T2WI/FLAIR (blue arrow; tumors typically appear hyperintense on T2WI and FLAIR image due to their different water content compared to surrounding brain tissue), hypointense on T1WI (blue arrow), showing minimal diffusion restriction on DWI and foci of blooming on SWI, suggesting hemorrhage adjacent perilesional edema is noted. The hypointensity on T1WI is suggestive of high-grade glioma (tumors in the CG often appear as hypointense areas on T1WIs). (1B) The lesion is causing a mass effect in the form of effacement of adjacent sulcogyral spaces, the frontal horn, and the body of the lateral ventricle with displacement of the falx toward the right, compressing the body and the isthmus of the CC. CC, corpus callosum; CG, cingulate gyrus; DWI, diffusion-weighted imaging; FLAIR, fluid-attenuated inversion recovery; SWI, susceptibility-weighted imaging; T1WI, T1-weighted image; T2WI, T2-weighted image

A region that appears different from the surrounding tissue on an MRI scan (Figure 1A) is referred to as a signal intensity that has been altered by heterogeneously intense enhancement. The area looks brighter on the T2-weighted image (T2WI) and fluid-attenuated inversion recovery (FLAIR) image when there is hyperintensity on T2WI/FLAIR (blue arrow). This might indicate the existence of edema or specific pathologies like tumors or inflammation. When an area looks darker on T1-weighted images (T1WI), it is said to be hypointense on T1WI (blue arrow). A high-grade glioma, a form of brain tumor, may be the cause of this observation. Minimal diffusion restriction on diffusion-weighted imaging suggests that there is only a small amount of water diffusion restriction in the region, which may be a sign of a less aggressive or less cellular tumor. Focuses of blooming on susceptibility-weighted imaging suggest that hemorrhage next to perilesional edema is noted. This shows that there are areas of magnetic susceptibility, probably because blood products are present in those places. In the setting of hemorrhage, which can be a hallmark of some brain tumors, this observation is frequently observed.

Figure 1B shows the mass effect, which refers to the pressure that the lesion is placing on the surrounding brain tissue. The lesion causes the normal spaces and structures within the brain, such as the sulci and gyri, the frontal horn, and the body of the lateral ventricle, to collapse or compress. This is referred to as the effacement of adjacent sulcogyral spaces, the frontal horn, and the body of the lateral ventricle. The natural anatomy of the brain may be altered as a result of this. The falx is a fold in the dura mater that divides the two hemispheres of the brain. The falx is being pushed to the right by the lesion, according to its displacement. The CC is a structure that links the two cerebral hemispheres. Compression of the CC’s body and isthmus indicates that the lesion is pressing against this crucial connecting channel.

Therapeutic interventions 

Specific goals for rehabilitation are addressed by the patient’s treatment plan. Proprioceptive Neuromuscular Facilitation (PNF) Rhythmic Initiation procedures are used, with 10 repetitions over three sets to be done three times daily, to improve the range of motion among the right proximal and distal limbs. To normalize tone in the affected limbs, the Roods Facilitatory Approach is also used in conjunction with tapping, icing, and quick range of motion exercises three times per day for three repetitions across three sets. Active assisted range of motion exercises are used to maintain the existing range of motion. They are performed 10 times in three sets, three times per day. The patient also receives multimodal sensory stimulation, which involves visual, auditory, tactile, and olfactory stimuli, for a period of 30 minutes to an hour, three times a day, to raise their degree of consciousness. The patient’s overall prognosis and functional capacity are projected to gradually improve as a result of the collective adoption of these therapies (Table 4).

**Table 4:** Phase I The above treatment is given for the first and second weeks. PNF, Proprioceptive Neuromuscular Facilitation

Goal	Intervention	Dosage and prognosis
To improve range of motion	PNF Rhythmic Initiation (passive range of motion) for the right upper limb and lower limb (Figure 2)	Ten repetitions × three sets (thrice a day)
To normalize the tone	Roods Facilitatory Approach for the right upper and lower limb tapping, icing, and quick range of motion	Three repetitions × three sets (thrice a day)
To maintain a normal range of motion	Active assisted range of motion for the right upper and lower limb	Ten repetitions × three sets (thrice a day)
To improve the level of consciousness	Multimodal sensory stimulation (visual, auditory, tactile, and olfactory)	Thirty minutes to one hour (thrice a day)

**Figure 2:**
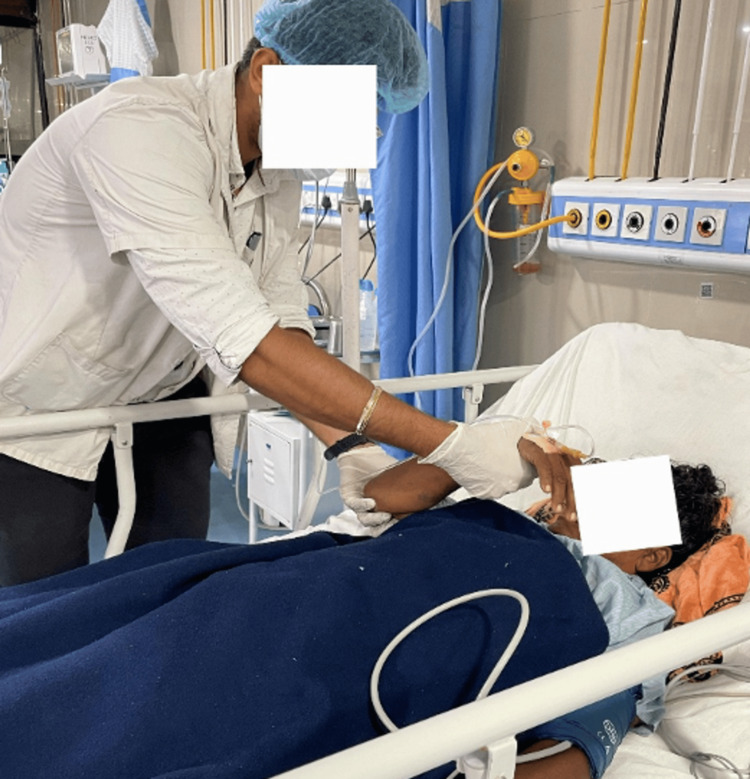
Performing the PNF pattern (D1 flexion) for the upper limb PNF, Proprioceptive Neuromuscular Facilitation

The rehabilitation program was created to focus on particular objectives for the patient’s recovery. PNF methods are used to increase range of motion, beginning with rhythmic initiation and moving on to active assisted range of motion and passive range of motion exercises. Three times a day, 10 repetitions total over three sets, are to be put into these exercises. The intervention also emphasizes increasing bed mobility through a variety of exercises, such as rolling on a mat, and transfer activities, such as switching from supine to side lying, side lying to sitting, and eventually advancing to sit-to-stand motions. These exercises must be done three times per day, starting with five repetitions and increasing to 10 as time goes on (Table 5).

**Table 5:** Phase II The above treatment is given for the third and fourth weeks. PNF, Proprioceptive Neuromuscular Facilitation

Goals	Intervention	Dosage
To improve range of motion	PNF Rhythmic Initiation progresses to active assisted range of motion and then passive range of motion for the right upper limb and lower limb (Figure 3)	Ten repetitions × three sets (thrice a day)
To improve bed mobility	Mat activities: rolling; transfer activities: supine to side lying, side lying to sitting, and sit to stand	Five repetitions progress to 10 repetitions (thrice a day)

**Figure 3:**
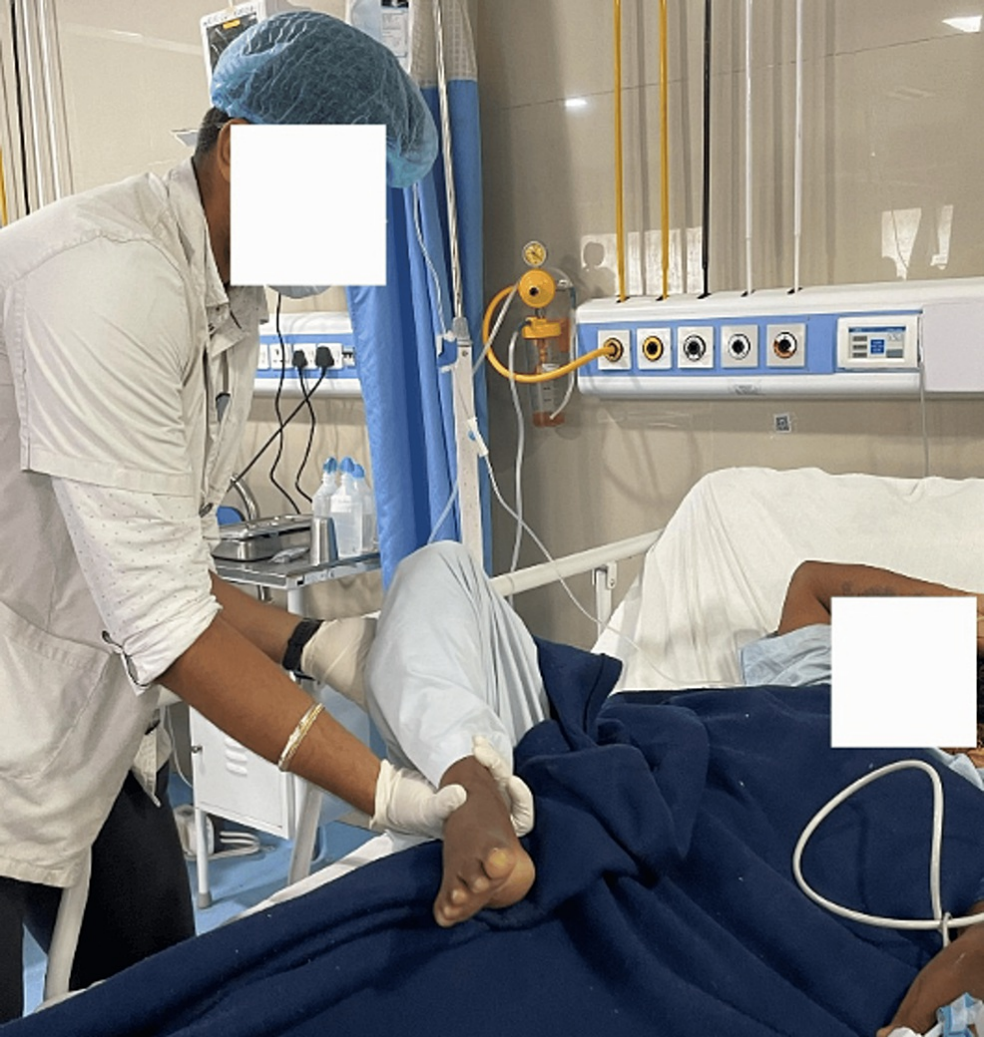
Performing the PNF pattern (D2 flexion) for the lower limb PNF, Proprioceptive Neuromuscular Facilitation

The goal of the patient’s rehabilitation program (Table 6) is to improve a few key physical abilities. The program includes dynamic quadriceps exercises that start as active assisted motions and advance to fully active movements, as well as isometric workouts that target the quadriceps, hamstrings, and back muscles. Each exercise is done three times a day for a total of 10 repetitions, with a five-second hold at first, increasing to a seven-second hold, and subsequently 10-second holds. The program includes exercises like sit to stands within a pain-free range of motion, single-leg stands, and tandem walking to improve balance. The repetitions for these exercises start at five, and they increase to 10, to be done three times per day. Additionally, to enhance gait, the regimen calls for spot marching exercises and parallel bar gait training with virtual and verbal feedback. For best results, the patient’s general mobility and functional ability should be efficiently increased by performing these gait exercises three times per day. The overall objective of the targeted interventions is to improve the patient’s physical performance and well-being by fostering strength, balance, and gait.

**Table 6:** Phase III The above treatment is given for the fifth and sixth weeks.

Uses	Exercise	Intensity
To improve strength	Isometric workouts for quadriceps, hamstrings, and back, followed by dynamic quadriceps exercises with active assistance and then progress to fully active dynamic exercise	Five-second hold progresses to seven-second hold, then 10-second with 10 repetitions × three sets
To improve balance	Sit to stand in a pain-free range of motion, single-leg stand, and tandem walking	Initially, at five repetitions, then progress to 10 repetitions
To improve gait	Spot marching and gait training with virtual and verbal feedback on parallel bars	Thrice a day

Outcome measures

The outcome measurements (Table 7) are a reflection of how the patient’s progress was assessed both during and after therapy. The GCS score of E1, V1, and M1 initially indicated little ocular, vocal, and motor responses in the patient. After therapy, the GCS score of E4, V4, and M6, indicating a noticeably higher degree of consciousness and responsiveness, was obtained, indicating a considerable improvement.

The patient’s performance on the Berg Balance Scale for balance showed a significant improvement, going from a pre-treatment score of 0 out of 56 to a post-treatment score of 40 out of 56, indicating a significant improvement in the patient’s capacity to maintain balance and stability. The patient also made significant progress on the Functional Independence Measure, with a pre-treatment score of 0 out of 126, indicating a high level of dependency on carrying out activities of daily living. The patient’s post-treatment assessment showed a significant improvement, with a score of 98 out of 126, suggesting improved functional independence and an increased capacity to complete a variety of everyday tasks with little help. Collectively, these post-treatment results reflect the intervention program’s beneficial effects on the patient’s cognitive function, balance, and overall functional independence.

**Table 7:** Outcome measures BBS, Berg Balance Scale; FIM, Functional Independence Measure; GCS, Glasgow Coma Scale; KPS, Karnofsky Performance Scale; mRS, modified Rankin Scale

Outcome measures	Pre-treatment	Post-treatment
GCS	E1, V1, and M1	E4, V4, and M6
BBS	0/56	40/56
FIM	0/126	98/126
mRS	4	3
KPS	50	80

## Discussion

In this case report, we delve into a rare instance of physiotherapy intervention in a patient diagnosed with glioma of the CG. The study presents a unique opportunity to review and analyze the outcomes of physiotherapeutic approaches tailored specifically for individuals with this rare brain tumor. By focusing on the reported results, we aim to elucidate the significance of physiotherapy in addressing the challenges posed by CG gliomas, shedding light on its potential impact on motor function, coordination, and overall quality of life for affected individuals. This analysis serves as a valuable exploration into the intersection of physiotherapy and the management of CG gliomas, offering insights that may contribute to our understanding of effective rehabilitation strategies in this uncommon clinical context.

The recent observations reveal atrophic alterations of the posterior CG in the presence of generalized atrophy of the whole brain (i.e., increased ventricular-brain ratio and decreased brain volume), CC, and thalamus in traumatic brain injury participants, lending credence to the claim of widespread damage. GCS was linked with CG atrophy, apart from the cognitive outcome. Different factors contributing to the absence of a connection between CG atrophy and neuropsychological performance are examined in the following sections [19-20]. It is noteworthy to mention right away that posterior CG atrophy was the main factor contributing to the overall reduction of CG surface area in patients. The front-to-back fiber track arrangement in the CG is less centralized than it is in the CC, where it coincides with the frontal-to-occipital anatomy [21]. After surgery, pain emerges as a prevalent concern among patients, and physiotherapy is crucial in resolving these issues. Important frontotemporal connections, including hippocampal input, are organized posteriorly with the CG due to the increased risk of hippocampus atrophy and frontal and temporal damage from trauma [22-24].

Considering the patient’s subjective experience is equally paramount. Any reported improvements in the patient’s quality of life, subjective well-being, or perceived benefits from physiotherapy were highlighted. Conversely, challenges and limitations encountered during the intervention were acknowledged, contributing to a more holistic understanding of the therapeutic process. By dissecting the outcomes, we gain insights into the effectiveness of the physiotherapeutic methods employed, potentially establishing a foundation for future interventions in similar cases. Additionally, the discussion delves into the specific physiotherapy techniques utilized in this case. This involved exercises targeting motor skill enhancement, coordination drills, and interventions aimed at addressing any pain or discomfort associated with the glioma. The rationale behind the chosen interventions, their frequency, and their duration are critical components for understanding the comprehensive rehabilitation plan.

## Conclusions

This report of a rare case on the physiotherapy intervention for a patient diagnosed with a glioma of the CG offers valuable insights into the tailored management of this unique neurological condition. The reported outcomes underscore the potential efficacy of physiotherapeutic strategies in addressing the multifaceted challenges associated with CG gliomas. The observed improvements in motor function, coordination, and overall functional abilities suggest that a carefully crafted and individualized physiotherapy approach can contribute positively to the well-being of patients with this rare brain tumor. The significance of this case report extends beyond its singular instance, providing a foundation for future research and clinical considerations. The specific physiotherapy techniques employed, their rationale, and the patient’s subjective experience offer valuable lessons for healthcare professionals working with similar cases. Moreover, the discussion prompts a critical reflection on the adaptability of physiotherapeutic interventions in the context of CG gliomas, potentially informing the development of standardized rehabilitation protocols for this unique patient population.

While this report adds to the limited literature on physiotherapy interventions for CG gliomas, it also highlights the need for further research to establish evidence-based practices in this domain. The rarity of such cases underscores the importance of collaborative efforts among healthcare professionals to accumulate knowledge, share experiences, and refine therapeutic approaches for improved patient outcomes. In essence, this case report serves as a valuable stepping stone in understanding the role of physiotherapy in the management of CG gliomas. By emphasizing the reported successes, challenges, and broader implications, this study contributes meaningfully to the ongoing discourse surrounding rehabilitation strategies for individuals facing this uncommon neurological condition.
